# Racial Disparities in Breast Cancer Genetic Testing May be Mitigated by Counseling

**DOI:** 10.1245/s10434-024-15434-2

**Published:** 2024-05-29

**Authors:** Julie B. Siegel, Melanie Bertolino, Rupak Mukherjee, Kiersten Meeder, Kevin S. Hughes, Andrea M. Abbott

**Affiliations:** Department of Surgery, Medical University of South Carolina, Charleston, SC USA

**Keywords:** Breast cancer, Genetic counseling, Racial disparities, Genetic testing, Genetics

## Abstract

**Background:**

Currently, racial disparities exist in access to genetic testing. Recent developments have helped narrow the gap in accessibility. The purpose of this study was to determine whether racial disparities in genetic consultation attendance and completion of genetic testing persist, and, if so, factors that contribute to under-utilization of these resources.

**Methods:**

A single-institution retrospective review of breast patients referred for genetic counseling between 2017 and 2019 was performed. Univariate and multivariate logistic regression evaluated factors associated with genetic counseling attendance and genetic testing.

**Results:**

A total of 596 patients were referred for genetic counseling: 433 (72.7%) white; 138 (23.2%) black; and 25 (4.2%) other or unknown. In multivariate analysis, black patients, patients without breast cancer family history, and patients without a current cancer diagnosis, classified as high risk, were significantly less likely to attend their genetics appointment (*p* = 0.010, *p* = 0.007, *p* = 0.005, respectively). Age, insurance type, distance from facility, and need for chemotherapy did not significantly impact consult completion rate. Of the patients who completed a genetic consult, 84.4% (*n *= 248) had genetic testing and 17.7% (*n* = 44) had a pathogenic variant. For patients who attended counseling, there were no significant factors that were predictive with receipt of genetic testing.

**Conclusions:**

In this study, there was a significant association between race and attending genetic counseling. Once counseled, most patients went on to receive genetic testing, and racial disparities in testing disappeared, emphasizing the value of providing additional education about the importance and purpose of genetic testing.

Breast cancer is the second-leading cause of cancer-related death for women in the United States.^1^ It is estimated that 10% of these breast cancer diagnoses are attributed to pathogenic variants.^2^ More than 50% of these pathogenic variants are secondary to high penetrance genes, such as BRCA1 and BRCA2, which confer a 50–85% lifetime likelihood of developing breast cancer.^2,3^ Knowledge of this risk is imperative, because it alters surveillance, surgical interventions, and therapeutic recommendations for patients and their families. For example, patients and relatives may be offered prophylactic surgery because of their increased risk of breast and ovarian cancer.^4^ Having an increased risk of breast cancer also alters screening recommendations. Patients with a lifetime risk of ≥ 20% receive recommendations to undergo annual breast MRI and to begin screening 10 years before the age of diagnosis of the affected family member.^5^ If patients identify that they have a pathologenic variant, predisposing them to breast cancer, they would have increased surveillance to identify cancers earlier. Recommendations on which patients should be tested for pathogenic variants differ between cancer and breast societies. The NCCN recommends testing for patients diagnosed with breast cancer ≤ 50 years and those at high risk for other reasons while American Society of Clinical Oncology recommends testing for patients diagnosed with breast cancer ≤ 65 years or those at high risk for other reasons.^5,6^ The American Society of Breast Surgeons have the most inclusive recommendations; they published an updated consensus on genetic testing in 2019 stating that all patients with a personal history of breast cancer or with a strong family should be offered genetic testing.^2^ Therefore, there exists some variability on how breast surgeons and oncologists refer patients.

The past decade has seen an increase in the accessibility of genetic testing. Before a Supreme Court decision in 2013, there was only one commercially available test for identifying BRCA pathogenic variants.^3^ Now, there are multiple commercially available tests, many of which are multigene panels that identify pathogenic variants that confer risk for breast cancer and other malignancies. The advent of next-generation sequencing also has significantly decreased the cost of these multigene panels.^3^ Multigene panels include a combination of high, moderate, and low penetrance genes.^3^ Counseling or informed consent is utilized to help navigate the results of genetic testing and to explain the benefits and risks of genetic testing for patients.^4^

Historically, there have been racial disparities in receipt of BRCA genetic testing.^7^ Decreased rates of genetic testing have been demonstrated in racial minorities with lower rates of genetic testing for high risk due to family history seen among black patients compared with white patients.^7,8^ The increased availability of genetic testing, along with expanded criteria for genetic testing, might mitigate barriers to genetic testing, and we aimed to investigate whether disparities in genetic counseling and testing exist after patients receive a physician referral for genetic counseling services. The literature has shown a multitude of other factors contributing to disparities in genetic testing, such as insurance type, previous knowledge about genetic testing, and increased concern by minorities on the use of genetic testing information.^9^ Expanded access to genetic testing may not mitigate all of these factors; we hypothesized that disparities continue to exist even when patients are appropriately screened and referred from a breast cancer expert.

## Methods

### Data Source and Variables

In this single-institution study, all patients who were referred for a genetic counseling appointment because of a personal or family history of breast cancer were included for analysis. Referrals were placed by the provider during the initial consultation when patients met genetic testing guidelines, and the provider recognized that to be true. All patients with breast cancer were presented at the weekly multidisciplinary tumor board, and meeting guidelines for genetic testing were discussed, which allowed patients to be referred if they were previously missed during initial consultation. Patients are not referred if they have already undergone genetic testing or if they refuse referral. A query of the electronic medical record was performed utilizing one or more specified ICD10 codes associated with family history of breast cancer or breast pathology during the years 2017–2019. This specific pre-COVID-19 time period was chosen due to two factors: (1) presence of an on-site genetic counselor, and (2) significant changes were made to genetic testing practices during the COVID-19 pandemic, which may have contributed to variations in genetic referral and testing that cannot be accounted for within the parameters of this study. During the time period investigated in this study, there was no charge or co-pay for the genetic counseling visit. No-shows were contacted three times via phone and then sent a letter. The genetic counselor at the time of this study identified as Asian descent. This study was reviewed by the institutional review board and was found to be exempt.

Patient characteristics were obtained from the electronic medical record. Variables extracted included: age, sex, race, distance from hospital, insurance type, receipt of magnetic resonance imaging (MRI), breast diagnosis, breast procedure, receipt of chemotherapy, family history of breast cancer, attendance at genetics appointment, receipt of genetic testing, and diagnosis of genetic mutation (pathogenic variant, VUS, no variant). For analysis, some variables were coded into two or more categories. Race was coded into white, black, other, and unknown. Distance from the hospital was coded for analysis into three categories: < 12.5 miles, 12.5–50 miles, and > 50 miles (these distance categories have been used in previous studies that have evaluated the effects of distance on healthcare outcomes^10^). Insurance type was coded into three categories: private insurance and Medicare, Medicaid, and self-pay. Breast diagnosis was coded into benign breast disease, ductal carcinoma in situ (DCIS), invasive breast cancer, and family history of breast cancer only. Degree of breast cancer family history was coded to any first degree relative, only second degree relative, only other degree relative, no family history, and unknown. Breast procedure was coded into none, lumpectomy, mastectomy, and bilateral mastectomy.

All statistical analyses were completed using the STATA statistical software package (version 15.0, College Station, TX). To determine the effect of variables on the primary outcome, attendance at a genetics appointment, we fit a univariate logistic regression model. The dependent variables used in this model included: age, race, distance traveled, insurance type, breast diagnosis, family history, and receipt of chemotherapy. The regression coefficients were estimated and odds ratios and 95% confidence interval were computed. Variables with *p*-values < 0.10 in univariate analysis were entered into the multivariate logistic regression model. A *p*-value < 0.05 was considered statistically significant. This analysis was repeated with the dependent variable, receipt of genetic testing, for the subset of patients that attended their genetic counseling appointment. For patients that had a pathogenic variant, we tabulated the procedures they had by race.

## Results

During the study time period from 2017 to 2019, our institution’s cancer center had 1654 new breast cancer referrals. A total of 596 patients was referred for genetic counseling because of an increased risk of breast cancer or a personal history of breast cancer during the study period (Table 1); 97.3% (*n* = 580) were women, and the average age at referral was 52.2 (STD ± 13.9) years. Most patients were white (72.7%, *n* = 433), and nearly a quarter were black patients (23.2%, *n* = 138). This is consistent with our institution’s cancer care population. Other minority races accounted for less than 2% of the study population. There was a nearly equal distribution on how far patients traveled for their care with 37.9% traveling < 12.5 miles (*n* = 226), 28.5% traveling 12.5–50 miles (*n* = 170), and 32.9% traveling > 50 miles (*n* = 196). The majority of patients had either private insurance or Medicare (*n* = 526, 88.3%). Almost half of the patients (46%, *n* = 274) were referred because of family history of breast cancer and did not have a current or past diagnosis of breast cancer. Forty-six percent of patients had a diagnosis of DCIS or invasive cancer (DCIS 6.4%, invasive 40.1%). In the cohort, 49.3% of patients (*n* = 294) reported a first degree relative with breast cancer while 18.1% of patients (*n* = 108) did not have any family history.

**Table 1:** Patient demographics

Patient characteristics (*N* = 596)	Number (%)
Female	580 (97.3)
Age (average [SD])	52.2 (13.9)
*Race*	
White	433 (72.7)
Black	138 (23.2)
Asian	6 (1.0)
Other	14 (2.3)
Unknown	5 (0.8)
*Distance (mi)*	
< 12.5	226 (37.9)
12.5–50	170 (28.5)
> 50	196 (32.9)
Missing	4 (0.7)
*Insurance*	
Medicare/private	526 (88.3)
Medicaid	59 (1.00)
Self-pay	11 (0.02)
MRI	224 (37.6)
*Breast diagnosis*	
Family history only	274 (46.0)
Benign	44 (7.4)
DCIS	38 (6.4)
Invasive cancer	239 (40.1)
Lymphoma	1 (0.2)
*Family history*	
First-degree relative	294 (49.3)
Second-degree relative	148 (24.8)
Other relative	34 (5.7)
No family history	108 (18.1)
Missing	12 (2.0)
*Breast procedure*	
Lumpectomy	148 (24.8)
Mastectomy	81 (13.6)
Bilateral mastectomy	81 (13.6)
No breast procedure	286 (48.0)
Chemotherapy	80 (13.4)
Attended genetics appointment	294 (49.3)
Genetic test	248 (41.6)
*Mutation*	
Variant of unknown significance	77 (12.9)
Pathogenic variant	44 (7.4)

**Fig. 1:**
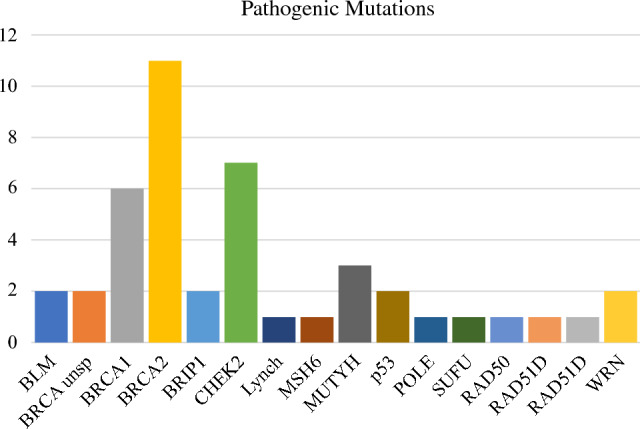
Distribution of pathogenic mutations in the study sample

The rate of genetic counseling appointment attendance after referral was 49.3% (*n* = 294). Eighty-four percent (*n* = 248) of patients who attended the genetic counseling appointment completed genetic testing. A pathogenic variant was diagnosed in 44 patients (7.4% of the total study sample) (Fig. 1), and 77 patients (12.9%) were found to have a VUS. Among pathogenic variants, the most frequent were BRCA2, 25% (*n* = 11), CHEK2, 15.9% (*n* = 7), and BRCA1, 13.6% (*n* = 6). Of the 44 patients with pathogenic variants, six (13.6%) were black; 50% of these patients had BRCA pathogenic variants.

Univariate logistic analysis was conducted to identify factors associated with attendance at a genetic counseling appointment (Table 2). In univariate analysis, not having a personal diagnosis of DCIS or invasive cancer significantly decreased the odds of attending a genetic counseling appointment. Having a positive family history and receipt of chemotherapy were positive predictive factors of attending a genetic counseling appointment. There was a trend toward significance in univariate analysis for black patients being significantly less likely to attend their genetic counseling appointment (OR 0.684, 95% confidence interval (CI) 0.465–1.01, *p* = 0.054).

**Table 2:** Factors associated with attendance of genetics appointment

Patient characteristic	Univariate analysis OR (95% CI)	*p*	Multivariate analysis OR (95% CI)	
Age	1.01 (0.998–1.02)	0.118		
*Race*				
White	Base		Base	
Black	0.684 (0.465–1.01)	0.054	0.577 (0.379–0.878)	0.010*
Other	0.611 (0.245–1.52)	0.29	0.637 (0.237–1.71)	0.371
Unknown	0.229 (0.025–2.07)	0.189	0.229 (0.024–2.21)	0.203
*Distance (mi)*				
< 12.5	Base			
12.5–50	1.02 (0.683–1.52)	0.931		
> 50	0.773 (0.526–1.13)	0.188		
*Insurance*				
Self-pay	Base			
Medicaid	1.24 (0.341–4.52)	0.743		
Medicare/private	1.16 (0.351–3.86)	0.804		
*Breast Dx*				
DCIS	Base			
Invasive cancer	1.06 (0.526–2.14)	0.869	1.06 (0.501–2.26)	0.872
Family history	0.451 (0.085–0.558)	0.024*	0.352 (0.169–0.735)	0.005*
Benign	0.217 (0.225–0.902)	0.002*	0.201 (0.075–0.538)	0.001*
*Family history*				
No family history	Base			
First-degree	1.17 (0.751–1.83)	0.484	2.05 (1.21–3.48)	0.007*
Second-degree	1.79 (1.08–2.95)	0.023*	2.82 (1.59–5.01)	< 0.001*
Other degree	4.55 (1.89–10.97)	0.001*	6.61 (2.55–17.1)	< 0.001*
Unknown	1.4 (0.424–4.622)	0.581	2.71 (0.762–9.64)	0.124
Chemotherapy	1.98 (1.21–3.22)	0.006*	1.39 (0.803–2.40)	0.24

*p < 0.05

In multivariate analysis, black patients were significantly less likely to attend their genetic counseling appointment compared with white patients (OR 0.577, 95% CI 0.379–0.878, *p* = 0.010). A positive family history continued to be a positive predictive factor for attendance (*p* < 0.05). Having only a family history (without a diagnosis of breast cancer) or a benign breast diagnosis also continued to be negative predictive factors for attendance of a genetic counseling appointment in multivariate analysis. Receipt of chemotherapy lost significance in multivariate analysis as a predictor of appointment attendance (Table 2).

Factors associated with receipt of genetic testing were analyzed for patients that attended their genetics appointment (Table 3). In univariate analysis, increased age was significantly associated with receipt of genetic testing (OR 1.02, CI 1.00–1.05, *p* = 0.018), and patients with a benign breast diagnosis were significantly less likely to have genetic testing performed (OR 0.080, CI 0.008–0.835, *p* = 0.035). There were no significant differences among race categories for receipt of genetic testing in patients who attended their genetic counseling appointment. In multivariate analysis, there were no factors that were significantly associated with receipt of genetic testing.

**Table 3:** Factors associated with genetic testing

Patient characteristic	Univariate analysis	*p*	Multivariate analysis	
	OR (95% CI)		OR (95% CI)	
Age	1.02 (1.00–1.05)	0.018*	1.02 (0.994–1.04)	0.139
*Race*				
White	Base			
Black	0.838 (0.386–1.82)	0.654		
Other	0.285 (0.065–1.25)	0.096		
*Distance (mi)*				
< 12.5	Base			
12.5–50	1.17 (0.557–2.45)	0.681		
> 50	1.55 (0.703–3.41)	0.278		
Insurance type		0.912^a^		
*Breast Dx*				
DCIS	Base			
Invasive cancer	0.350 (0.044–2.77)	0.32	0.401 (0.050–3.20)	0.388
*Family history*	0.167 (0.021–1.30)	0.087	0.223 (0.027–1.80)	0.159
Benign	0.080 (0.008–0.835)	0.035*	0.098 (0.009–1.06)	0.056
Family history				
No family history	Base			
First-degree	0.603 (0.215–1.69)	0.337		
Second-degree	0.818 (0.265–2.52)	0.727		
Other degree	0.417 (0.113–1.53)	0.188		
Unknown	0.625 (0.060–6.49)	0.694		
Chemotherapy	2.47 (0.843–7.22)	0.1		

^a^Fisher’s exact test used for univariate analysis due to groups present with ≤ 5 observations

*p < 0.05

For patients that had a pathogenic variant, we tabulated the breast procedure obtained by race (Table 4). There were 37 white patients and six black patients with a pathogenic variant identified in this study. Bilateral mastectomy was performed in 11 patients, all were of white race; two of these were for bilateral breast cancer. No statistical analysis was conducted as the study was underpowered to detect statistically significant differences.

**Table 4:** Comparison of breast procedure by race for patients with pathologic mutations

Patients with pathogenic mutation (*N* = 44)	Race	
	White (*N* = 37)	Black (*N* = 6)
*Breast procedure*		
None	16 (40.0)	2 (40.0)
Lumpectomy	7 (18.9)	1 (16.7)
Mastectomy	5 (13.5)	2 (33.3)
Bilateral mastectomy	11 (29.7)	0 (0)

## Discussion

Pathogenic variants can alter management for patients with or without breast cancer.^11,12^ In this study, we identified that despite referral to genetic counseling, only 49.3% of patients attended their appointment. Notably, once patients attended their appointment, they were likely to proceed with genetic testing. This finding suggests that the barrier to testing is the requirement for a separate visit to a different professional.

Counseling itself was associated with a high rate of genetic testing. In the present study, significant, independent variables associated with not attending a genetic counseling appointment included not having a family history of breast cancer or not having a personal history of breast cancer (i.e., only having a family history of breast cancer) and black race. Interestingly, we found that travel distance, insurance type, and age were not significant factors in attendance of the genetics appointment. When analyzing factors associated with receipt of genetic testing, once an appointment was attended, there were no variables that were significant barriers to genetic testing. Because not all patients who returned for a counseling appointment received genetic testing, there are likely barriers that were not elucidated in the analysis. For example, most patients had some form of insurance, and the self-pay subset was too small to accurately analyze this variable. For those with insurance, there may be variability on specific insurance plans and coverage for genetic testing. Furthermore, patients may not be aware of what their insurance covers and may be wary of the potential costs.

Although access to genetic testing has been improved by lower cost, previous studies have shown that genetic testing rates are still poor.^12–15^ Evaluating our entire sample, the rate of genetic testing was 41.6% of those referred. This rate is higher than some of the recent literature. A retrospective cohort study of the Georgia and California Surveillance, Epidemiology and End Results (SEER) databases from 2013 to 2014 evaluated genetic testing rates for patients with breast and ovarian cancer and found that only 24.1% of breast cancer patients had genetic testing completed.^12^ They found that age, poverty, and less aggressive disease showed decreased genetic testing rates, but they did not see decreased testing rates by race or ethnicity.^12^ The higher rates that we observed in the present study may be secondary to continued, improved access to genetic testing and counseling and greater advocacy of testing at our facility compared with the average hospital represented in the SEER database. It is possible that it may be falsely elevated if referral orders were not placed appropriately; however, patients are seen in clinic multiple times during their breast cancer care and discussed at the multidisciplinary tumor board, so we would expect that if the referral was missed during a visit, it would have been placed at a subsequent visit. Rates of genetic testing after genetic counseling were high at 84.4%, suggesting that education and advisement on genetic testing may be a significant contributor to receipt of testing.

Previous studies have shown that black patients are less likely to undergo genetic testing.^5^ A qualitative study by Sheppard et al.^16^ that used focus-group methodology explored barriers to genetic counseling and testing for black women with either a personal history of breast cancer or a first-degree relative with breast cancer; the study identified that little knowledge on counseling and testing seemed to contribute to low utilization of these services. The study also demonstrated that fear was a barrier, more predominantly in women without a personal history of breast cancer, and concern for discrimination was also a voiced concern for all women in the study.^16^ Similarly, a cross-sectional survey of awareness and attitudes about genetic testing demonstrated lower awareness and less belief in benefits of genetic testing in black women. Survey results also showed an increased concern of racial discrimination from genetic testing among black women.^17^ In the present study, once black women attended their genetic counseling appointment, they were not less likely than other races to complete their genetic testing. This suggests that counseling may mitigate some of these barriers, such as lack of knowledge, fear of the unknown, and concern for discrimination. Further investigation is warranted to determine how counseling can be increased for patients.

Increasing rates of counseling and thus genetic testing can have significant implications for black patients. Epidemiology studies have revealed that black women have higher rates of BRCA 1 and 2 pathogenic variants in comparison to their non-Hispanic white counterparts.^18,19^ Knowledge of these pathogenic variants may allow these patients to undergo prophylactic surgeries or have earlier and more intensive (i.e., addition of MRI) surveillance, increasing survival in this population of patients.

Increasing rates of genetic counseling may need greater allocation of resources to bolster awareness, increase education, and decrease fear for patients. Rates of genetic counseling also might increase merely by additional advisement by the physician. A population-based study found that the difference in rates of BRCA1/2 testing among black women were largely attributed to physician recommendations.^20^ Thus, the role of physicians as educators and advocates for their patients regarding genetic testing may need to be expanded. However, physicians may already be providing these services, and patients may forget some of this information in the time between their breast appointment and their genetics appointment. If resources permit, genetic counseling services during the breast clinic visit could increase overall testing completion rates. In 2021, Rana et al.^21^ observed the impact of embedding a genetic counselor into oncology clinics on rates of genetic testing. Before implementation, 66% of ovarian cancer patients referred for genetic testing were tested.^21^ After a genetic counselor was embedded into the clinic workflow, 80% of women referred for genetic testing completed their testing.^21^ Many breast clinics are now utilizing a multidisciplinary model whereby patients are scheduled to see a surgeon, oncologist, and radiation oncologist during their appointment. Integrating a genetic counseling appointment would be a logical addition to the multidisciplinary care of the breast cancer patient. Point-of-care genetic testing, if a counselor is unavailable, also could increase rates of genetic testing. Currently, in response to the low overall rates of genetic testing, the surgeon and advanced practice providers, at our institution, are testing patients in the clinic, and we will be analyzing how this affects are genetic testing rates. Chai et al.^22^ instituted a mainstream genetic testing where patients were counseled and tested by their surgeon during their clinical encounter. Incorporating this model significantly increased the rates of genetic testing and decreased wait times for genetic testing.^22^ Mainstreaming genetic testing within the clinic appointment has also shown high rates of genetic testing in the ovarian cancer population in several studies from the United Kingdom.^23–25^ Members of the cancer team, who are not genetic counselors, receive training for consenting patients for genetic testing and incorporate it during the initial clinical consultation.^25^ Patients are notified of the genetic results by these providers consenting the patients, and then they are referred to additional counseling based on their result.^25^ These studies have shown 95–100% uptake of genetic testing.^23–25^ There may be some socioeconomic and cultural factors in the United Kingdom that differ from those in the United States, and more specifically in the southeast United States, where this current study was conducted, but there are still likely benefits of mainstreaming that are generalizable. In the United States, the impact of point-of-care genetic testing has been demonstrated in Whitworth and colleagues^26^ study evaluating the effects of large national healthcare insurance payers instituting a mandate that requires genetic counseling before testing. These mandates significantly increased cancellations of genetic testing, and cancellations rates showed sharper increases in the Latin American and African American subgroups.^26^ With mandates potentially in place for some patients, point-of-care testing might not be feasible at all institutions that do not have the resources to provide same day counseling in addition to testing. Even if a genetic counseling mandate is not a barrier, there may be practices that do not have the time or the staffing to provide point-of-care genetic services. Tele-counseling has shown to be equally efficacious as traditional genetic counseling for increasing rates of genetic testing.^27^ Although it may not abolish a second appointment for testing, using virtual genetic counseling would allow a means by which education on genetic testing could be provided and mitigate some of the barriers of genetic testing. Success in virtual counseling suggests that there may a role for genetic counseling videos, chatbots, and/or artificial intelligence. Using chatbots as adjuncts for distributing genetic testing information and consenting patients is still in its infancy, but the current literature shows that patients opinions’ on this tool is favorable.^28^ There also are some benefits to chatbots that patients voice including giving patient’s more information during the consent process and giving patients more time to think about their questions.^28^ However, patients also voice concerns about privacy using these tools.^28^ With point-of-care testing showing improvement in rates of genetic testing, this may be a solution for a lack of personnel to distribute information. This would be another resource that cancer centers would need to invest in, but they may eventually see return of this investment. If resources permit, point-of-care genetic services has been shown to increase genetic testing for patients and also might mitigate the racial disparities.

This study was conducted at a single institution, which may limit the generalizability of the results. Patient demographics, insurance, and clinical workflow may vary at other institutions and may be influenced by geographic location. A larger sample size would increase the statistical power of this study and allow more insight into the differences in genetic testing across populations and among different races and ethnicities. Furthermore, other confounding factors that may impact patients’ decisions to undergo genetic testing were not accounted for, including education level and cultural and religious beliefs. These factors might play a significant role in patient attitudes toward genetic testing and surgical management of disease. Our sample was fairly homogenous in terms of insurance type, with most patients having an insurance type that would have covered genetic testing, which limits the ability of this study to fully assess the influence of socioeconomic factors on genetic counseling and testing. Further understanding of other relevant variables would provide a more comprehensive understanding of the complexities involving genetic counseling and testing in different populations.

## Conclusions

The findings of this study have significant implications for clinical practice related to breast cancer management. It is important for breast cancer patients to explore genetic counseling and testing to identify pathogenic variants that can help to facilitate personalized management and surveillance. Detection of variants has the potential to alter management decisions and decrease morbidity and mortality related to breast cancer. The results of this study suggest that barriers previously seen for genetic testing are still present, and disparities in genetic testing may be mitigated by point of care counseling and testing. Disparities in genetic testing may stem from the inconvenience of a second visit or lack of knowledge regarding the importance and purpose of genetic testing. Further resources may need to be allocated to increase patient education around genetic counseling and testing to increase utilization of these services.

## Data Availability

Kevin Hughes, MD, has founded and has a financial interest in CRA Health (Formerly Hughes RiskApps), which was acquired by Volpara in January 2021. CRA Health develops risk assessment models/software with a particular focus on breast cancer and colorectal cancer. Dr. Hughes is the Co-Creator of Ask2Me.Org, which is freely available for clinical use and is licensed for commercial use by the Dana Farber Cancer Institute and the Massachusetts General Hospital (MGH). Dr. Hughes’s interests in CRA Health and Ask2Me.Org were reviewed and are managed by Massachusetts General Hospital and Mass General-Brigham in accordance with their conflict of interest policies. None of the other authors have conflict(s) of interest with the content of this manuscript.
